# The nature of grief: implications for the neurobiology of emotion

**DOI:** 10.1093/nc/niae041

**Published:** 2024-12-17

**Authors:** Matthew Ratcliffe, Pablo Fernandez Velasco

**Affiliations:** Department of Philosophy, University of York, York YO10 5DD, United Kingdom; Department of Philosophy, University of York, York YO10 5DD, United Kingdom; Institute of Behavioral Neuroscience, University College London, 26 Bedford Way, London WC1H 0AP, United Kingdom

**Keywords:** emotional experience, emotional processes, grief, regulation, scaffolding

## Abstract

This paper explores the limitations of neurobiological approaches to human emotional experience, focusing on the case of grief. We propose that grief is neither an episodic emotion nor a longer-term mood but instead a heterogeneous, temporally extended process. A grief process can incorporate all manner of experiences, thoughts, and activities, most or all of which are not grief-specific. Furthermore, its course over time is shaped in various different ways by interpersonal, social, and cultural environments. This poses methodological challenges for any attempt to relate grief to the brain. Grief also illustrates wider limitations of approaches that conceive of emotions as brief episodes, abstracted from the dynamic, holistic, longer-term organization of human emotional life.

## Introduction

The aim of this article is to identify some methodological constraints on the neurobiological study of emotions by considering the nature of grief. These constraints are not so apparent when emotions are conceptualized as discrete, decontextualized, short-term responses to significant events in one’s environment. But grief poses a challenge in virtue of (i) its temporally extended structure, and (ii) how its trajectory depends upon social scaffolding. Our point is not merely that grief cannot be approached in a certain way or that certain questions cannot be sensibly asked of grief. In addition, there is the prospect of adopting grief as an exemplar for thinking about human emotional life more generally. Even where other emotions do take the form of brief episodes, these are often—although not always—embedded in temporally extended, socially scaffolded patterns that are inextricable from the ever-changing organization of one’s life. Hence, a consideration of grief points to the need for a wider perspectival shift.

In fact, there has been surprisingly little work on grief and the brain. As O’Connor (2019, p. 8) remarks in a recent survey of relevant literature, “the neurobiology of grief is still in its infancy.” Indeed, we arrived at the topic of this paper when—as part of a larger project on grief—we decided to investigate what had been written about its neurobiology. Most of us experience grief at some point in our lives, and it is one of the most profound, enduring, and self-affecting emotional experiences we will ever face. So, unsurprisingly, it is a major topic across many disciplines, including psychology (Bonanno and Kaltman 2001), anthropology (Silverman et al. 2021), and—in recent years—philosophy (Ratcliffe 2023). Expecting to face a mountain of literature on the neuroscience of grief, we were instead surprised to find so little and began to wonder why that might be. Furthermore, most of the research that has been undertaken has a more specific focus. For instance, there are studies addressing neurobiological differences between typical and pathological forms of grief, how grief relates to illness, and individual differences in the ability to self-regulate during grief (e.g. Freed et al. 2009, O’Connor 2019, Kakarala et al. 2020).

In light of this sparse literature, it might seem that there is little need for critical discussion. However, in addition to affecting us profoundly, grief is associated with a range of health outcomes (Fagundes and Wu 2020). Hence, for practical as well as theoretical reasons, it is important to determine how neuroscience can inform our approach to grief. As things stand, the paucity of discussion in neuroscience is at odds with the wider importance of the topic. By considering which questions it makes sense to ask about grief and the brain, we can also draw attention to larger methodological issues concerning the neuroscientific study of emotions. Despite various disagreements over the nature of emotions and how they relate to the brain, it is frequently assumed that emotions are—at least for the most part—discrete episodes that depend largely or wholly on different combinations of neural mechanisms. For example, many emotion researchers have endorsed one or another account of “basic emotion,” according to which basic emotions of different types are associated with different physiological changes, feelings, and expressions (e.g. Ekman 2003, Panksepp 2005). Others have suggested that different emotions depend on many of the same neural mechanisms but put together in different ways, so that a type of emotion need not be associated with a dedicated neural architecture (Scherer 2005, 2009). (For a review of theories of emotion in neuroscience, see Hamann 2024). An added complication is that definitions of “emotion” also vary. For example, although different emotional feelings are often taken to feature in different emotions, Le Doux (1999, p.12) instead identifies emotions with “biological functions of the nervous system” and maintains that feeling is not part of the emotion itself but one’s consciousness of that emotion.

However, grief is neither a discrete episode nor a longer-term state. Instead, it unfolds over lengthy periods and incorporates many different emotional experiences. A grief process, we will suggest, is not reducible to however many episodes that might be abstracted from the whole and accounted for wholly in terms of associated neurobiological mechanisms. Furthermore, its temporal course depends in various ways on interpersonal, social, and cultural scaffolding. This constrains what can be learned about grief by turning to the brain, perhaps accounting for the paucity of research in this area. It is further arguable that what applies to grief applies to human emotional experience more generally—it is dynamic, holistic, temporally extended, and regulated by patterned interactions with social environments. While the term “grief” usually refers to the emotional process as a whole, other such emotional processes may lack established names. Instead, we refer to the many different emotional episodes that together comprise them. But here too, our understanding remains impoverished if we attend exclusively to isolated episodes while neglecting larger dynamic patterns, how those patterns relate to the changing structures of our lives, and how they are embedded in social environments.

To develop our position, we begin by offering an overview of existing studies of grief in neuroscience. Following this, we defend the view that grief should be understood as a socially scaffolded process and set out the challenges this conception entails for the neuroscientific study of grief. Finally, we draw some conclusions and propose future directions for the interdisciplinary study of grief.

## The neuroscience of grief

A functional magnetic resonance imaging (fMRI) experiment by Gündel et al. (2003) may well have been the first neuroscientific study of grief. Eight recently bereaved women were shown a photograph combined with a word. The experiment followed a 2 × 2 (person-by-word) factorial design: Participants compared a photograph of the deceased, which they had provided, with a photograph of a stranger (person factor) and personalized grief-related words with matched neutral words (word factor). This resulted in four conditions (deceased photograph, grief word; deceased photograph, neutral word; stranger photograph, grief word; stranger photograph, neutral word), each consisting of 15 picture-word composites. Emotional responses to the grief-related stimuli were intense but very brief, as measured by changes in skin conductance. The picture and word factors independently activated the posterior cingulate cortex, the medial/superior frontal gyrus, and the cerebellum. Subsequent studies have similarly shown the posterior cingulate cortex, which is part of the network involved in autobiographical memory, to be active during grief elicitation tasks (O’Connor et al. 2007, Schneck et al. 2018, Jain et al. 2019). Of course, these regions subserve a wide variety of processes and are not specific to grief. The same regions are said to be involved in affective processing, automatic motor responses, autonomic regulation, mentalizing, and visual imagery (Critchley et al. 2003, Maddock et al. 2003, de Borst et al. 2012, Romano et al. 2020, Arioli et al. 2021).

Several other studies have employed a similar grief elicitation paradigm. One of these found a relationship between autonomic arousal and posterior cingulate activity during grief elicitation (O’Connor et al. 2007). A subsequent study by O’Connor et al. (2008) looked at the activation patterns of recently bereaved women. The experimenters selected 23 participants and conducted clinical interviews with them (based on Prigerson and Jacobs 2001), which led to eleven of the women being diagnosed with “complicated grief.” (The proposed diagnostic category “complicated grief” has since been largely superseded by “prolonged grief disorder”, although there is considerable overlap between the two diagnoses. See, e.g., Prigerson et al. (2021) for further discussion.) O'Connor and colleagues found that, following the presentation of grief-related stimuli, only those with complicated grief diagnoses showed activation in the nucleus accumbens, an important area for reward processing. The experimenters interpreted this activation of the reward network as a neural instantiation of painful yearning, a craving-like response toward the deceased on the part of those with complicated grief. This result, while suggestive, has not been replicated (McConnell et al. 2018).

Other neuroimaging studies have adapted emotional and cognitive versions of the Stroop task (e.g. to examine attentional bias involving words relating to the deceased). In the emotional Stroop task, participants are asked to name the font color (e.g. green) of words with either neutral (e.g. table) or emotional (e.g. shame) valence. The task is generally used to measure attentional bias toward emotional stimuli. One study using this task found increased activation of the rostral anterior cingulate cortex (associated with emotion regulation) for individuals with noncomplicated grief, compared to both those who had not suffered bereavements and those diagnosed with complicated grief (Arizmendi et al. 2016). Another study found that prefrontal-amygdala connectivity (regulatory and saliency regions, respectively) correlated with deceased-related attentional bias (Freed et al. 2009). However, a later study using an emotional and a cognitive Stroop task found that neural activation patterns were similar for deceased-related stimuli and for stimuli relating to those still living (Schneck et al. 2018).

Other methodological approaches include volumetric analyses, pharmacological interventions, and machine learning. Concerning volumetric analyses, decreased brain volume has been associated with complicated grief (Saavedra Pérez et al. 2015). In addition, parents who lost their only child were found to have decreased left hippocampal volumes (Luo et al. 2016), and those who had experienced “affective loss” (which covers both bereavement and breakups) were found to have a larger amygdala, larger nucleus accumbens (for men), and smaller hippocampus (Acosta et al. 2021). A well-known limitation of this approach is the difficulty of reliably inferring causal connections from structural differences in cross-sectional volumetric studies (Thomas and Coecke 2023). Regarding pharmacological interventions, a recent study attempted to change resting state connectivity patterns via oxytocin administration in recently bereaved participants (Seeley et al. 2023). The experimenters identified a network pair (default-retrosplenial and cingulo opercular-dACC) in which higher resting state functional connectivity was associated with fewer symptoms of prolonged grief. They also found that intranasal oxytocin increased functional connectivity in that same circuit. However, the effect of oxytocin was not moderated by prolonged grief symptoms (contrary to their hypothesis). Finally, an fMRI study by Schneck et al. (2017), involving a grief elicitation and a sustained attention task, identified a neural pattern of activation in the insula, basal ganglia, and orbitofrontal cortex that corresponded to thoughts about the deceased in multiple modalities. They then used neural decoding, a two-step machine learning technique to detect a target cognitive process (in this case, deceased-related thoughts) based on neural data. They trained the decoder on a set of the fMRI data, and the decoder was then able to predict which patterns of the patient’s brain activity correlated with thoughts about the deceased on a different set of fMRI data.

Although neuroscientific studies of grief are fairly scarce, a picture is starting to emerge after 20 years of work, involving a conspicuous lack of robust findings. Key findings have not been replicated, the evidence for differential activation of key areas (e.g. the anterior cingulate cortex in complicated grief) is equivocal, and attempts to measure overall levels of neurotransmitters associated with complicated grief symptoms have been inconclusive (Kakarala et al. 2020). O’Connor (2019), a leading researcher in the area, acknowledges this lack of decisive, replicated findings. As potential explanations, she suggests that the tasks involved (such as Stroop or grief elicitation) may not be ideal, that diagnostic criteria for complicated grief might not be reliable, and that the sample sizes of previous studies were not large enough. However, while these may well be important considerations, we contend that the issue goes deeper. It lies in the very nature of grief, which is a multifaceted, temporally extended, socially scaffolded process that cannot be reduced to its various components.

## Grief as a process

Many approaches to grief share the assumption that it is neither a string of disparate emotional episodes nor an enduring mood. Instead, grief consists in an organized process of some sort. For example, Kübler-Ross and Kessler (2005) famously—and controversially—proposed that a grief process involves five overlapping stages: denial; anger; bargaining; depression; and acceptance. However, conceiving of grief as a process need not involve any commitment to discernible stages. A pressing issue for any account of grief as a process is that of what, if anything, unites its various constituents, such that they comprise a unified whole rather than a disparate, heterogeneous sequence of other emotional experiences, thoughts, and activities (Goldie 2012). Any claims concerning “grief and the brain” will be undermined by a lack of clarity over the nature of grief. For instance, if grief is a temporally extended, integrated process, then questions such as “what are the neural correlates of grief?” are poorly formulated, as such a process could potentially encompass the full range of human emotions.

Determining whether grief is a process need not involve appealing to empirical evidence. In fact, it is plausibly incoherent to think of grief as occurring at a moment in time, rather than over time. This is not an empirical matter; it concerns shared, pre-reflective understandings of grief, which tend to be presupposed rather than explicitly challenged by scientific enquiry. In his *Philosophical Investigations*, Wittgenstein (1953, p. 174) offers this well-known remark: “‘For a second he felt violent pain.’—Why does it sound queer to say: ‘For a second he felt deep grief’? Only because it so seldom happens?” He goes on to suggest that feeling grief now is akin to playing chess now. The comparison is an illuminating one. To elaborate on Wittgenstein’s remark, we can slip in and out of a game of chess or begin a game of chess and eventually give up. But imagine someone who only ever played momentary chess, moving a Knight on one board never to return, moving a pawn on another board a few days later, and so forth. It would eventually become apparent to us that this person is not playing chess at all; their actions are insensitive to norms that are constitutive of the game of chess.

But why should momentary grief be similarly incoherent? Granted, there are no strict norms of grief. Even so, grief is not just something we experience in response to a death. Over time, emotional engagement with the death “does something,” which cannot be accomplished in an instant, given that it requires interacting with and altering one’s social environment. The person who has died may have been integrated into one’s life in many important ways—into habitual activities and patterns of thought, expectations, projects, goals, commitments, and enduring values. Hence, coming to comprehend or—if you like—fully “believe” that they have died is not just a matter of accepting the proposition “that person is dead” and other propositions implied by it. The whole organization of one’s life might need to change radically in order to accommodate what has happened, including habits and expectations that permeate pre-reflective experience—this is their room, the sofa where we sit, their place at the breakfast table, our car, the park where we play, the café where I meet them every Sunday morning. Initial acceptance of the proposition “that person is dead” can remain at odds with the structure of one’s life, including patterns of engrained expectation that still point to the person’s potential presence and to various ways in which one might interact with them. Read (2018) has thus proposed that there is an irreducibly temporal “logic” to grief, incorporating experiences of tension, disbelief, unreality, and the like. We cannot overturn our habitual expectations in an instant; they need to be repeatedly dashed and brought into conflict with our explicit, linguistic beliefs.

This process extends to how we experience the surrounding world as a whole. For the most part, our surroundings are experienced as mattering to us in fairly consistent, organized ways—the arrangement of items on my desk that appear to me as a significant whole in light of a writing project, the park where we enjoy walking the dog, the train that takes me to work in the morning, and so forth. How a situation matters to us reflects our various projects, pastimes, and habits. Without a particular person, many of these can become unsustainable—we did this together; I did this for her; this made sense in relation to our future plans. Hence, a world that was once taken for granted as a backdrop to our experiences, thoughts, and activities is undermined by the death. Our response therefore involves what Thomas Attig (2011) has called “relearning the world.” This can span all aspects of one’s life—habits, expectations, projects, plans, goals, commitments, and values are all reconfigured to varying degrees. What unifies various constituents of grief is that they together contribute to the process whereby one’s world comes to accommodate, at least to some degree, the fact of the death (Ratcliffe 2017, 2022). This is why the notion of grieving for a moment and then simply stopping, without leaving anything undone, turns out to be incoherent. When another person is integrated into our biography, our habits, and our expectations to such an extent, we cannot fully accept the proposition “they are dead” in an instant. Doing so requires interacting with the surrounding world and reorganizing our life over a period of time.

For the same reasons, it would be a mistake to think that we could identify the neurobiological underpinnings of grief by focusing on what is experienced at any given moment. Of course, one could respond by suggesting that grief is, after all, reducible to its various shorter-term components. Either way, though, any straightforward claim concerning how “grief” relates to specific neural correlates or brain processes is undermined. Suppose we identify neurobiological correlates of A, where A is integral to a longer-term process B. This need not tell us anything of interest about B. It could be that A is similarly integral to emotional processes C, D, E, and F, which are precisely what we seek to distinguish from B. For example, O’Connor (2005) discusses the fMRI study conducted by Gündel et al. (2003), involving a “grief-eliciting paradigm” where photographs and words were used to induce emotional responses among the bereaved. Although O’Connor acknowledges that this only gives us only a “snapshot” of grief (the responses measured via skin conductance were very brief), it is questionable whether it provides even that. The type of emotional experience elicited at that time could potentially contribute to many other kinds of emotional experience too. If so, studies like this reveal nothing of what makes grief distinctive. The brain regions identified by the study in question are involved not only in grief but also in affective processing more widely, in the retrieval of emotion-laden episodic memories, in autonomic regulation, in visual imagery, and in mentalizing. More generally, there is a risk of posing questions that presuppose implausible conceptions of grief. For example, Peña-Vargas et al. (2021, p. 1) take grief to be one of seven “primary emotions” and to consist of an “unwanted and unpleasant feeling.” They also refer to “activation of the grief neurological pathway.” However, if grief is instead conceived of as a temporally extended, heterogeneous process, it makes no sense to think in terms of the grief pathway. (However, see also O’Connor and Seeley 2022, p. 317) for a discussion of grief and neurobiology that does acknowledge the central importance of a lengthy re-learning process, as well as the tensions that are involved between habitual expectations and the reality of one’s situation).

One might object at this point that we sometimes do experience what at least seem to be “moments” of grief. For instance, long after someone has died, we might have a profound but fleeting feeling of loss, perhaps elicited by a particular place, situation, or artefact. Although it is unclear whether or how such experiences participate in larger processes, we still face the same methodological problems. Suppose we accept that a token experience is not—in any informative sense—part of a preceding grief process. It remains unclear how we could study grief by somehow eliciting such experiences. If what we are studying is indeed just a momentary emotional experience, then there is—as yet—no evidence to indicate that any such experience is unique to grief. But now suppose instead that the experience includes more than just that—one’s current emotions, recollections of past emotions, autobiographical memories, a sense of presence or absence, feelings of interpersonal connection, and so forth. If that is so, then such experiences have a complex, heterogeneous structure and are also likely to encompass considerable variety. For those reasons, they are unlikely to involve a distinctive, consistent pattern of neural activation.

To make matters even more difficult, the acknowledgement that bereavement can undermine one’s world should not be taken to imply that grief is occupied solely with this. Our concern for someone who has died and what they have lost need not be exhausted by the task of reorganizing our own habits, projects, commitments, and expectations, even if the two are inextricable (Ratcliffe 2020). It can be added that a need to “relearn” the world is not specific to bereavement. Illness, injury, impairment, unemployment, relationship break-ups, cultural upheaval, migration, and a host of other circumstances can all undermine one’s world in structurally similar ways (Cole and Ratcliffe 2022, Ratcliffe and Richardson 2023). This raises a further methodological problem—the referent of the term “grief” is unstable (even if we accept that grief always takes the form of a temporally extended process). Grief is sometimes understood specifically as a response to bereavement, but we also talk of grief in various other circumstances—over the children we never had, the place we can never return to, the person they were before the accident. So, even where there is something informative to say about the neurobiology of grief (as opposed to an emotional experience that just happens to be integral to a particular grief process at a certain time), the scope of the claim can be unclear—what kind(s) and circumstances of grief are we referring to? For example, Freed et al. (2009, p. 34) investigated the brain systems involved in grief by recruiting subjects who had recently lost a pet. They maintain that pet loss is accompanied by “symptoms” that are “analogous” to grief over the death of a person. However, even if that is so, the potential limits of the analogy require clarification. It could be that, in some cases, pet loss similarly undermines the habitual organization of a life. Nevertheless, the question remains of whether and to what extent it approximates the experience of losing a person. We thus face the additional task of determining whether what is learned from pet loss applies specifically to bereavement grief or, alternatively, to grief in a broader sense. It may also apply to some bereavement and/or nonbereavement losses but not to others. And, of course, “pet loss” could itself encompass a diverse range of grief experiences.

In summary, if—as seems plausible—grief is a process that cannot be reduced to its various components, neurobiological research needs to start by acknowledging the following: (i) it is difficult to make any informative generalizations about grief and the brain; (ii) studies of grief are sometimes better construed as studies of grief’s constituents, which are not specific to grief; (iii) informative claims about components of grief do not add up to informative claims about grief; and (iv) the scope of claims about grief requires clarification. Taken together, these four observations plausibly account for the current dearth of robust findings; it is not clear what the various studies are actually addressing or whether different studies are addressing the same thing. As we will now see, another important consideration stemming from a process conception of grief is how the structure of grief depends not only on combinations of neurobiological mechanisms but also, to a significant degree, on structured interactions with various forms of environmental “scaffolding.”

## Scaffolding for grief

If grief is a temporally extended process, what makes it distinctive (phenomenologically and more generally) need not be a consistent emotional quality or grief-specific type of emotional episode. Instead, we might look for a temporal “pattern” (Goldie 2012) or “logic” of grief (Read 2018). We have proposed that this consists—at least in part—of a tension-riddled process whereby one comes to reconcile the propositional belief “that person is dead” with one’s changing experiential world or life structure. How such processes unfold over time does not depend exclusively on the internal psychology of an individual. It also depends to a large extent on how the interpersonal, social, and cultural environment is organized. Grief, we might say, is “scaffolded” in a number of different ways by our social surroundings. For current purposes, the term “scaffolding” refers to how an environment is organized and acted upon in order to change the tasks faced by one or more individuals (Sterelny 2010). There is growing acknowledgement of the extent to which human emotional life involves scaffolding—how we manipulate our surroundings in order to elicit, alter, or extinguish emotional experiences in ourselves and others (Colombetti and Krueger 2015, Krueger and Osler 2019, Coninx and Stephan 2021). The concept of scaffolding applies to both episodic emotions and longer-term moods. However, it is important to further acknowledge the distinctive way in which emotional processes such as grief depend on scaffolding.

A grief process is fragile and precarious, as the events that elicit grief “pull the rug from under our feet,” so to speak. Ordinarily, events, entities, and situations are experienced as mattering to us against the backdrop of a dynamic life structure that incorporates numerous interconnected habits, expectations, projects, goals, and values. This structure serves to elicit, prescribe, constrain, and regulate patterns of activity, including activities directed toward regulating our emotional experiences. For instance, we might listen to music, go for a swim in the sea, visit an art gallery, seek consolation from a friend, or drink coffee. It is arguable that, insofar as both our social environment and the more specific organization of our own life remain fairly stable, the majority of our emotional experiences do not require separate regulatory processes at all. Instead, they take care of themselves or “auto-regulate” as we act in ways that change our relationship with a practically meaningful environment (Kappas 2011). For example, our fear of the approaching car prompts us to get out of the way, after which it no longer appears frightening. Nothing more is required in order to regulate our fear. The point applies similarly to social interactions. For instance, our being visibly upset will sometimes influence the actions of others in such a way that they cease to cause us distress.

In contrast, the death of another person can disrupt the organizing background against which more mundane emotional experiences arise and unfold. To varying degrees, the structure of one’s life is lost before new structure can be established. With this, there is a pervasive sense of indeterminacy, disorientation, lack of direction, or being “lost” (Ratcliffe 2017, 2020, 2022, Mehmel 2023). This is not merely a matter of “not knowing” what to do. The very basis for one’s choices is eroded; the projects, relationships, commitments, habits, and pastimes relative to which those choices made sense and could be judged rational have themselves become unsustainable. In an intriguing first-person account of grief, the neurologist Lisa Shulman emphasizes that grief is more a matter of profound disorientation than of characteristic feelings of sadness and the like; it involves “waking up each day in an unfamiliar world where all rules are scrambled” (2018, p. 45). (For further discussion of orientation and disorientation, see, for example, Stegmaier (2019), Fernández Velasco et al. (2021), and Mehmel (2023)). Shulman further remarks on the importance of being open to new possibilities and, with this, to the prospect of reorganizing one’s life and identity. At the same time, though, she maintains that the experience of grief is to be understood in terms of brain processes: “our emotional life and attachments […] are generated by a vast network of neural transmission and signaling”; “all of our emotions and behavior, indeed the totality of our experience, has a neurologic basis” (2018, p. 66, p. 91). In light of this, Shulman suggests that studies of the brain have a crucial role to play in furthering our understanding of grief.

This sort of brain-centric talk also features in many other discussions of emotion. For example, Le Doux (1999, pp. 9–10) refers to “how emotions come from the brain,” “how the brain detects and responds to emotionally arousing stimuli,” and how the brain “makes us happy, sad, afraid, disgusted, or delighted.” Panksepp (2005, p. 162) even refers to the emotional feelings that “brains experience,” thus suggesting that the human brain—as opposed to the human organism interacting with the social environment—is the subject of emotional life. However, once we take into consideration grief’s process structure and what it is that grief does, we are prompted to look further afield. Grief involves engaging, over a lengthy period of time, with the disturbance of a life structure that would otherwise contribute to shaping and regulating one’s emotional experience. Importantly, grief itself is therefore rendered fragile and precarious by this lack of embeddedness in a familiar world. Various established, dependable relations with one’s surroundings, which one might have drawn upon in other circumstances, are absent. The concept of “emotional scaffolding” is thus closely tied to that of “emotion regulation.” Not all emotion regulation requires emotional scaffolding, but all emotional scaffolding can be construed in terms of emotion regulation. And the task of regulating grief is complicated by the loss of social scaffolding that was integral to one's capacity for emotion regulation (For further discussion of grief and emotion regulation, see Ratcliffe and Byrne (2022a). For wider discussion of conceptions of emotion regulation, and empirical research on emotion regulation, see Gross (2014)).

The predicament is compounded when bereavement involves losing the very person to whom one would otherwise have turned during times of upheaval. Even where that is not so, bereavements can disrupt relationships with other family members and friends, rendering sources of interpersonal scaffolding unavailable. Hence, grief can be doubly disorienting—one is deprived of mundane forms of scaffolding, as well as more exceptional sources of scaffolding that are called upon in certain challenging situations.

The structure and duration of grief depend to varying degrees on whether and how one is able to continue drawing upon interpersonal, social, and cultural resources in order to comprehend and negotiate a protracted disturbance of one’s world. These resources play a range of interrelated roles. For instance, retention of life structure can be important. Certain aspects of one’s life may have been relatively unaffected by bereavement. So, there remains the prospect of retreating to them in order to sustain meaningful, coherently organized activities and regulate one’s emotions. As Shulman (2018, p. 51) writes, “returning to work, returning to my customary roles as neurologist, educator, and researcher, was sustenance to me.” This can also serve as a basis from which to establish new life structure. In contrast, other social activities are essential to confronting and adapting to loss. Only by interacting with familiar social situations do we come to fully recognize that habitual activities and expectations no longer apply. Social interactions are also essential to the revision of projects and pastimes, and to the development of new ones (Ratcliffe 2022).

Another important way in which interpersonal, social, and cultural environments shape grief is by contributing to how we make sense of what has occurred, what we now face, what has happened to the other person, and what the future holds. For instance, we co-construct various narratives with others, which are themselves embedded in larger contexts of shared practice. Some of these narratives are ephemeral, assembled over the course of particular conversations and never to be repeated. Others are maintained, further disseminated, developed, and revised. Narratives can provide frameworks for interpretation that have the potential to shape one’s activities. In addition, they feed into interpersonal interactions that open up other regulative possibilities. This applies similarly to culturally established narratives and rituals, which provide ways of interpreting and conveying experiences, and prescribe norms of interaction (Walter 1996, Higgins 2013, Ratcliffe and Byrne 2022b). Such narratives can also contribute to how we make sense of grief itself. For instance, it is arguable that putting emotions into words has the potential to resolve and reshape emotional experience, by rendering inchoate experiences more determinate and, in so doing, enabling us to act upon them in new ways (e.g. Colombetti 2009, 2014, Ratcliffe 2022). Other people provide further scaffolding by offering possibilities for “delegation.” When one’s own life is bereft of guiding organization, such that one is directionless and unable to discern meaningful choices, there can remain the prospect of drawing on the organized lives of others, in a manner analogous to following a guide in order to navigate a wilderness.

The scope of interpersonal scaffolding is not limited to those who are still living. As documented by a fast-growing literature on “continuing bonds” with the dead, the course of grief depends to a large extent on whether and how we continue to experience and relate to those who have died (Klass et al. 1996, Klass and Steffen 2018). A sense of enduring connection, facilitated in many different ways by our interpersonal and material surroundings, can itself amount to scaffolding for grief. Conversely, certain bonds have the potential to disrupt or prolong grief (Ratcliffe 2022). Additionally, there is the possibility of technological advancements contributing to the affective scaffolding of grief, such as the use of “deathbots”—chatbots based on the digital footprint of the deceased (Krueger and Osler 2022, Fabry and Alfano 2024). All of these contributions interact with one another over time, together playing an important role in how grief is experienced at any one time and also over time.

Once grief is conceived of in this way, it becomes readily apparent that its course cannot be accounted for solely in terms of processes internal to the organism. O’Connor and Seeley (2022, p. 318) appreciate the role of learning in grief, maintaining that “the brain’s model of the world must change.” But they frame this model-updating view in internalist terms, according to which the challenge comes down to a conflict between internalized representations in “semantic knowledge” (the term they use to denote an enduring belief in the persistence of the bereaved) and in “episodic knowledge” (memory of the person’s death). However, the process further depends on various interactions between individuals and social environments. These draw upon shared interpretive and practical resources, accommodating considerable variety. An emphasis on scaffolding also has implications for how we think of “resilience” in grief (e.g. Bonanno 2009). Reliance on a certain form of scaffolding may render a person better able to cope in some situations but not others. Furthermore, whether bereavement and other situations deprive one of scaffolding for grief may, in some instances at least, be more a matter of contingent circumstances than of traits internal to an individual. It is therefore likely that “resilience” is itself highly context-sensitive. For similar reasons, any proposed distinctions between “typical” and “pathological” forms of grief should not be conceived of wholly—or even primarily—in terms of processes internal to individuals. Both the temporal shape and the duration of grief depend to a large extent on how an individual interacts with the interpersonal, social, and cultural world. In a given case, what is lacking in the surrounding social environment may prove more salient in accounting for, and indeed modifying, the path of grief over time than what is internal to the individual.

## Conclusions

We began with an overview of neurobiological approaches to the study of grief, following which we highlighted the methodological difficulties that arise when grief is construed as a temporally extended and socially scaffolded process. We have argued that many important aspects of grief cannot be understood solely by studying constituent experiences and their relationships to brain processes. Grief is not so much a matter of which emotions are experienced at a given time as of how various emotional experiences contribute to an unfolding, variably integrated process. It can be added that the significance of any apparent similarities and differences between emotional episodes depends on how and where those episodes fit into the larger whole. For example, a current feeling of sadness might be suffused with hope or, alternatively, with only the prospect of more sadness to come. Depending on which, and on where in a grief process the relevant experience arises, it could contribute to the process and its direction in altogether different ways. The importance of such subtle but important similarities and differences cannot be discerned by focusing on isolated emotional episodes, abstracted from their place in longer-term emotional patterns. Nor can it be discerned by scrutinizing anything that might occur in the brain at that particular time. A more holistic, dynamic perspective on human emotional life is required.

None of this is to deny that neurobiology has important roles to play in affective science. Even if it cannot capture what is distinctive about grief *per se*, neuroscience could still help us to distinguish different forms of grief and what they involve at different times. For instance, although the predominance of certain episodic neural activities does not serve to illuminate the nature and distinctiveness of grief, some activation patterns may still be more typical of one form of grief (e.g. prolonged grief disorder or a more specific variant of prolonged grief) than others. Nevertheless, our approach is consistent with a shift away from cross-sectional data and toward longitudinal studies. The mainstream methodological and analytical approaches in neuroimaging are aimed primarily at cross-sectional studies. There are technical difficulties with longitudinal neuroimaging, including irregularities between subjects and also single-subject level measurements that are noisy and sensitive to imaging artefacts (Skup 2010). However, there are new approaches that can help overcome these difficulties, such as using large quantities of data with each subject (Naselaris et al. 2021), normative modeling frameworks (Rehák Bučková et al. 2023), multi-echo fMRI (Lynch et al. 2020), and a model of reciprocal validation between longitudinal precision studies and cross-sectional population studies (Gell et al. 2024). Recently, longitudinal neuroimaging studies have made potential inroads into our understanding of trauma (Roeckner et al. 2021), social isolation (Lammer et al. 2023), and depression (Lynch et al. 2024). Similar approaches offer promising avenues for exploring the longer-term structure of grief. In such studies, special attention should be paid to the grief trajectories of the individuals involved (Nielsen et al. 2019, Bonanno and Malgaroli 2020).

As for the affective scaffolding of grief, the concerns we present here show how neuroscientific paradigms remain limited and need to be embedded within a broader ecological approach, at both the conceptual and the methodological level. This is consistent with a renewed effort toward an ecologically minded affective science that triangulates heterogenous methodologies, measures, tasks, and reports. For instance, there are important developments regarding the collection of longitudinal data “in the wild” (e.g. phone-based heart rate and respiratory rate measurement; Bae et al. 2022), which can be combined with more traditional daily diary, experience sampling, or ecological momentary assessment methods to study emotional phenomena in daily life (Kuppens et al. 2022). In addition, there are technological developments, such as Functional Near-Infrared Spectroscopy, which can help study brain activity outside the confines of the laboratory (Doherty et al. 2023). And, more generally, real-world methodologies can be used to contextualize and contrast lab-based brain imaging studies. Finally, there are important improvements in the tasks that can be used in laboratory settings to provide more ecologically valid stimuli (Vigliocco et al. 2023). Mobile neuroimaging approaches have shown promise in the neuroscientific study of social interactions and everyday activities (Quaresima and Ferrari 2019, von Lühmann et al. 2021, Stangl et al. 2023). Similarly, they could help facilitate a move from elicitation paradigms to exploring grief as it unfolds in a social environment.

An emphasis on the interpersonal, social, and cultural dynamics of grief also complements certain themes in cultural psychiatry and, more specifically, recent calls for an “ecosocial” or “cultural-ecosocial” perspective in psychiatry. For instance, Kirmayer (2019) proposes that, more generally, psychiatry needs to shift from a brain-centric approach toward one that “recognizes social predicaments as the central focus of clinical concern,” along with the ways in which our mental lives are “intrinsically social.” Certain symptoms, it is maintained, are shaped, regulated, and interpreted via processes that span brain, body, and social environment (Gómez-Carrillo and Kirmayer 2023). The perspective that we are advocating also has more specific implications for how we conceive of those forms of grief that fall under the diagnostic category “prolonged grief disorder,” recently adopted by both the ICD and the DSM classification systems (albeit with slightly different diagnostic criteria). Regardless of which terms and diagnostic criteria we might apply, exceptionally distressing, disruptive, and prolonged experiences of grief are not simply attributable to processes internal to individuals, abstracted from their interpersonal, social, and cultural environments. How grief is experienced at a particular time and also over time further depends on numerous regulatory processes that involve interacting with specific individuals, other people in general, shared social environments, and cultural resources. Any number of different factors, many of them external to the individual, could turn out to be most salient in accounting for the nature of a particular individual’s grief experience. The extent to which grief’s course depends on social scaffolding was made especially apparent by radical social restrictions imposed during the COVID-19 pandemic, which prevented many people from being with friends and family members before they died, and from subsequently engaging with the social world in ways that would otherwise have involved reorganizing their lives and relationships over time. With this, there were increased reports of a grief that was unchanging, frozen, or on hold, bereft of a temporal organization that is inextricable from relating to and interacting with the social world over time (Ratcliffe 2023).

Construing grief as a fragile, scaffolded process serves not only to complicate a fairly small body of work on the neurobiology of grief; it is also of much broader interest. By taking grief as an exemplar through which to conceptualize emotional experience, we come to see that an emphasis on brief emotional episodes, considered in abstraction from their place in a life, yields an impoverished view of human emotional experience. This is not to suggest that such a picture should be dispensed with altogether. Rather, it should be supplemented by an additional, more encompassing theoretical perspective. One might object that this requirement is limited to emotions such as grief, which take the form of socially scaffolded processes. So, it plausibly extends to some token instances of guilt, remorse, shame, and perhaps other emotions as well. However, it is doubtful that the majority of established emotion types take this form. Why is such an approach relevant when it comes to studying the likes of fear, anger, happiness, and so forth? However, this apparent limitation may well prove to be an artefact of our emotion talk. That we identify one emotion process as grief while referring to another such process in terms of fear, followed by disappointment, followed by anger, relief, regret, and forgiveness need not imply that the latter is any less unified. So, in focusing exclusively on various constituent emotions, we still risk eclipsing their place in larger processes and, with this, the structure of human emotional life.

It is thus arguable that an emphasis on emotional processes and their social scaffolding has much wider applicability. Even when we do experience a fleeting episode of fear, followed by joy, followed by relief, these emotions often participate in larger patterns and are regulated by the organization of our lives. This organization is itself essentially dynamic, ever-changing in subtle and sometimes more pronounced ways. Furthermore, emotions are often experienced as temporally organized—there is a coherence to how one emotional experience leads to the next and to how our emotions reflect our short- and longer-term engagement with changing situations. Hence, overly atomistic thinking also eclipses the dynamic phenomenology of emotion.

In light of this, neurobiological research on emotion seems overly preoccupied with swift, automatic processes that are triggered by transient, decontextualized stimuli. For example, Ekman (2003, p. 20) maintains that “emotions prepare us to deal with important events without our having to think about what to do,” and Scherer (2005, 2009) likewise construes emotions as rapid, urgent responses. Our suggestion is not that such approaches should be abandoned, alongside established taxonomies of emotion that include episodes of fear, sadness, joy, and the like. The point is that such work provides only a limited perspective on human emotion, which omits what is perhaps distinctive of the emotional: how emotional patterns involve discerning and navigating actual and potential changes in the dynamic life organization through which we encounter events, entities, and situations as significant in one or another way.

In conclusion, then, when it comes to the nature of emotional experiences such as grief, there is little to be said from a synchronic, episodic perspective. Any findings concerning emotional episodes are to be situated within a larger view of human emotional life and their conclusions limited accordingly. There is also a further need for approaches to emotion and the brain that investigate dynamic patterns and their dependence upon interpersonal, social, and cultural contexts, rather than discrete episode types and their neurobiological underpinnings.

## Data Availability

No new data were generated or analyzed in support of this research.
